# Gastrointestinal Effects on the Antioxidant and Immunomodulatory Properties of South African Fynbos Honey

**DOI:** 10.1155/2023/2553197

**Published:** 2023-11-24

**Authors:** Innocentia Botlhale Magoshi, Anwani Wendy Nekhumbe, Mohammed Auwal Ibrahim, June Cheptoo Serem, Megan Jean Bester

**Affiliations:** ^1^Department of Anatomy, University of Pretoria, Pretoria 002, South Africa; ^2^Department of Biochemistry, Ahmadu Bello University, Zaria, Nigeria

## Abstract

The Fynbos biome, Western Cape Province, South Africa, produces a unique honey from *Apis mellifera capensis*. The bioactivity of Fynbos (FB1-FB6) honeys and Manuka, unique manuka factor 15+ (MAN UMF15+) honey subjected to simulated *in vitro* digestion, was compared. The effect of each phase of digestion on the antioxidant properties and nitric oxide- (NO-) associated immunomodulatory effects was determined. The total phenolic content of MAN (UMF15+) was higher than that of FB honeys, and following digestion, the percentage bioaccessibility (BA) was 68.6% and 87.1 ± 27.0%, respectively. With the Trolox equivalent antioxidant capacity assay, the activity of FB1 and FB6 was similar to MAN (UMF15+) but reduced for FB2, FB3, FB4, and FB5 with a %BA of 77.9% for MAN (UMF15+) and 78.2 ± 13.4% for FB. The oxygen radical absorbance capacity of MAN (UMF15+) and FB honeys was similar and unaltered with digestion. In a cellular environment, using colon adenocarcinoma (Caco-2) cells, both undigested and the gastric digested honey reduced 2,2′-azobis-(2-amidinopropane) dihydrochloride- (AAPH-) mediated peroxyl radical formation. In contrast, following gastroduodenal digestion, the formation of reactive oxygen species (ROS) was increased. In murine macrophage (RAW 264.7) cells, all honeys induced different levels of NO which was significantly increased with digestion for MAN (UMF15+) and FB1. In LPS/IFN-*γ* stimulated RAW 264.7 macrophages, only undigested MAN (UMF15+) effectively reduced NO levels, and with digestion, NO scavenging activity of MAN (UMF15+) was reduced but increased for FB5 and FB6. In a noncellular environment, MAN (UMF15+), FB1, FB2, and FB6 scavenged NO, and with digestion, this activity was maintained. This study has identified that undigested and gastric-digested FB honey has antioxidant properties with strong potential anticancer effects following gastroduodenal digestion, related to ROS formation. MAN (UMF15+) had anti-inflammatory effects which were lost postdigestion, and in contrast, FB5 and FB6 had anti-inflammatory effects postdigestion.

## 1. Introduction

Globally, honey is one of the most reputable functional foods, used for nutritional and medicinal purposes [1]. Honey is a complex mixture of sugars (glucose and fructose) and water with minor components that include polyphenols, volatile compounds, and vitamins such as ascorbic acid, enzymes, organic acids, methyl glyoxal (MG), hydrogen peroxide (H_2_O_2_), amino acids, and minerals [2]. The type, composition, and concentration of these components are dependent on the bee species, the botanical and geographical origin, edaphoclimatic conditions, and collection factors including processing and storage [2, 3]. Polyphenols, MG, H_2_O_2_, and peptides such as bee defensin have been identified as the major bioactive components that are responsible for the antioxidant, anti-inflammatory, antimicrobial, and anticancer properties of honey [1, 4, 5].

Seraglio et al. [2] have reviewed the effects of simulated gastrointestinal digestion on the levels of minerals, polyphenols, MG, *α*-dicarbonyls, sugars, organic acids, and vitamin C, as well as the associated antibacterial, antioxidant, and anticancer activity. The researchers concluded that the minerals in honey are bioaccessible; however, more information on the bioaccessibility and bioavailability of other components of honey is required. The number of studies that investigate the effect of simulated digestion on the antioxidant properties of honey is few and is limited to studies on Brazilian bracatinga honeydew, manuka, and blossom honey of unknown origin [2]. In these studies, the trend was that with gastric digestion bioactivity was unchanged, while following duodenal digestion, bioactivity effects were either decreased or increased activity. Seraglio et al. [2] evaluated the %BA of individual polyphenols in Brazilian bracatinga honeydew honey. The %BA was between 93 and 220%, and the associated antioxidant capacity was between 60.33 and 100%. The polyphenols unique for manuka honey were stable with simulated digestion [6]. O'Sullivan et al. [7] reported, for manuka and several commercial honeys, that the antioxidant activity was reduced by 62%. In addition, digestion reduced antioxidant activity of organic honey by 80%, and the %BA of honey-associated vitamin C was reduced to 47% [8]. Few studies have investigated the cellular effects of digestion; generally, following digestion, cell viability was reduced [7], and for manuka honey, this was identified as an anticancer effect where several anticancer cellular indicators such as apoptosis were increased [8].

The effect of honey on pro- and anti-inflammatory cytokines has been reviewed by Navaei-Alipour et al. [9]. Several cell-based studies have been undertaken, and Timm et al. [10] reported that in immune cell lines, the release of interleukin (IL)-6 by Mono Mac 6 cells was stimulated by 1% honey solutions. Four percent of manuka, kanuka, and clover honey increased tumour necrosis factor—alpha (TNF-*α*) levels in monocytic THP-1 cells [11]. Treatment with thyme honey increased the expression of TNF-*α* and cyclooxygenase -2 (COX-2), associated with an increase in prostaglandin E2 (PGE2) in RAW 264.7 murine macrophages [12]. In cancer cell lines, manuka honey inhibited IL-6 release by AGS (human gastric adenocarcinoma) cells [13], whereas thyme honey inhibited IL-6 secretion and TNF-*β* activity by PC-3 (human prostate cancer) cells [14] and IL-6 in human breast cancer cell lines [15]. No studies could be found on the effects of digestion on the pro- and anti-inflammatory properties of honey and an aim of this study was to address this gap in knowledge.

Nevertheless, the anti-inflammatory effects of honey in animal models have been investigated and provide an indication of the consequence of digestion. In rat and mouse models, oral consumption of honey reduced the levels of TNF-*α*, IL-6, NO, PGE2, inducible nitric oxide synthetase (iNOS), COX-2, and vascular endothelial growth factor (VEGF). In gastric ulcer models, honey reduced the gastric mucosal malondialdehyde (MDA), IL-1*β*, IL-6, and TNF-*α* [16]. In a further study, manuka honey was also effective against chronic acetic acid-induced gastric ulcers, and the observed effect was due to antioxidant and anti-inflammatory effects with a significant reduction in mucosal MDA levels, TNF-*α*, IL-1*β*, and IL-6 and an increase in IL-10 levels [17].

Although immunomodulatory effects of honey in the duodenum are unknown, in rat models of breast cancer, Tualang and manuka honey reduced TNF-*α* expression [18], while in a neuroinflammation model, Tualang honey reduced neuroinflammation with a considerable decrease in IL-*β* and TNF-*α* [9]. This indicates that associated bioactive molecules are both bioaccessible and bioavailable. Although widely consumed according to Navaei-Alipour et al. [9], only four randomized control trials have been undertaken to investigate the beneficial effects of honey consumption. Generally, it was found that there was a reduction in the markers of inflammation. No study could be found that investigated the effects of the different phases of simulated digestion in cell models on NO formation or scavenging. The modulation of NO levels in the gastrointestinal tract (GIT) is an important consideration as the role of NO in the GIT is complex. Nitric oxide has a cytoprotective effect, while in diseased states, NO inhibition is beneficial. Low NO stimulates, while high concentrations inhibit gastric secretion [19]. In addition, increased NO associated with *Helicobacter pylori* infection can cause gastric luminal inflammatory changes [20]. Inflammatory diseases of the bowel are also associated with increased inflammation, and effective treatment is associated with the suppression of leukotriene synthesis, inhibition of IL-1 synthesis, and the scavenging of reactive oxygen species (ROS) and peroxynitrite. Therefore, induction of NO in healthy individuals has a cytoprotective effect; however, in several inflammatory diseases of the GIT, the scavenging of ROS and/or NO prevents peroxynitrite formation with the subsequent reduction in inflammation.

Manuka honey is produced by bees, from the nectar of *Leptospermum scoparium* [21]. This honey is considered as the gold standard in the assessment of the beneficial effects of honey. The unique manuka factor (UMF) correlates with the MG levels in this honey. However, the beneficial effects of manuka honey are not limited to MG and include several other bioactive molecules [2]. In contrast, information on other types of honey such as Fynbos honey on inflammation is lacking. Fynbos honey is a unique honey produced by *Apis mellifera capensis* (Cape honey bee) in the Fynbos biome of the Western Cape Province of South Africa. The Fynbos biome is a unique region with endemic flowering plant species, namely, the Cape reeds (*Restionaceae*), heath or Erica (*Ericaceae*) family, and the Protea (*Proteaceae*) family [22]. Some research on the antioxidant [23] and antibacterial [24, 25] activity on Fynbos honey has been undertaken but is limited. In addition, studies on the digestion of Fynbos honey are lacking. In this study, the effects of simulated digestion, the impact of pH, and the phase of digestion on the antioxidant and NO-mediated immunomodulatory properties of Fynbos honey were undertaken and compared with MAN UMF15+ honey.

## 2. Materials and Methods

### 2.1. Honey Samples

Six Fynbos (FB) honey samples (FB1, FB2, FB3, FB4, FB5, and FB6) were purchased from local beekeepers and farm stores in the Western Cape Province, South Africa. The samples were transported in air-tight containers to the laboratory at the University of Pretoria, South Africa. In this study, manuka honey, MAN (UMF 15+), was used as a control and was purchased from a local health shop. The physiochemical properties of these honeys were determined as described previously by Serem and Bester [23].

### 2.2. *In Vitro* Simulated Human Gastrointestinal Digestion

Static *in vitro* digestion that simulates human digestion was performed according to a modified method of Daglia et al. [26]. Initially, a gastric digestion stock solution of 20 mg/mL pepsin in 1 M HCI and pancreatic stock solution of 4 mg/mL pancreatin in 1 M NaHCO_3_ were prepared. The honey samples were then diluted to 90% (*v*/*v*) to achieve fluidity and subjected to *in vitro* GIT digestion. For gastric digestion (GD), the pH of the honey solution was decreased to pH 2 followed by the addition of 5 *μ*L of the prepared pepsin solution/mL honey. The gastric digest was then incubated in a water bath for 30 min at 37°C. Then, for further gastroduodenal digestion (GDD), the pH of the honey samples was adjusted to pH 7 followed by the addition of 5 *μ*L of the prepared pancreatin stock solution/mL honey. The mixture was further incubated in the water bath for 60 min at 37°C. For each phase of digestion, pH controls (no enzymes added) were included. To inactivate the enzyme reactions, the samples were heated in a water bath at 95°C for 5 min. Throughout the digestion process, volumes of various solutions that were added were monitored to calculate final % honey concentrations. Assays were then performed with honey solutions at 1 or 10% honey (*v*/*v*).

### 2.3. Total Polyphenolic Content (TPC) Assay

The total polyphenolic content was determined using the Folin-Ciocalteu reagent according to Amin et al. [27]. A volume of 10 *μ*L of a 10% (*v*/*v*) honey samples was added to the wells of a 96-well plate followed by 50 *μ*L Folin-Ciocalteu reagent and 50 *μ*L of 7.5% sodium carbonate. After incubation for 15 min at room temperature, absorbance was determined at 630 nm, and the data was expressed as mg gallic acid equivalents (mg GAE/100 g).

### 2.4. Trolox Equivalent Antioxidant Capacity (TEAC) Assay

The TEAC assay was based on a method by Awika et al. [28] where freshly prepared 2,2′-azinobis(3-ethylbenzothiazoline-6-sulfonic acid) cation (ABTS^+^) was reacted with 3 mM potassium peroxodisulfate to 8 mM ABTS. After a 12 h incubation in the dark, 290 *μ*L of this solution was mixed with 10 *μ*L of a 10% (*v*/*v*) honey, pH controls, or digests. After 30 min, absorbance was read at 734 nm, and the antioxidant activity was expressed as *μ*mol TE/g.

### 2.5. Oxygen Radical Absorbance Capacity (ORAC) Assay

The ORAC assay was determined according to a modified method of Ou et al. [29]. A concentration of 0.12 *μ*M fluorescein (16 mL) was mixed with 14.8 mM AAPH (4 mL) to prepare a working solution. A 5 *μ*L volume of a 1% (*v*/*v*) honey, pH controls, digests, or Trolox (0–800 *μ*M) was then mixed with 195 *μ*L working solution. The samples were well mixed, and the fluorescence was read every 5 min for 4 h at an excitation and emission wavelengths of 485 nm and 520 nm, respectively. The ORAC values were calculated by using the Microocal Origin 6.0 which measured the total area under the decay curve (AUC), and the results were expressed as *μ*mol TE/g.

### 2.6. Dichlorofluorescein Diacetate (DCFH-DA) Assay

To determine the effect of a 5% honey solution on ROS formation, a modified method of Blasa et al. [30] was used. Human colon adenocarcinoma (Caco-2) cells were plated at 2 × 10^3^ cells/100 *μ*L in a 96-well plate and cultured for 24 h at 37°C and 5% CO_2_. Thereafter, 50 *μ*L of 75 *μ*M DCFH-DA was added to each of the wells, and the plate was incubated for 1 h at 37°C. The medium was then removed from the wells, and the cells were washed once with 0.1 M PBS followed by the addition of 50 *μ*L of 10% (*v*/*v*) honey. pH controls or digests and 50 *μ*L of 7.5 mM of AAPH to the wells. Negative controls included cells exposed to PBS only, whereas positive controls included cells exposed to only AAPH and PBS. The change in fluorescence was read (BMG labtechnologies Offenburg, Germany) every 2 min for 1 h at an excitation and emission wavelengths of 485 nm and 520 nm, respectively. The gradient of change in fluorescence was measured, and the percentage oxidative damage (%OD) was calculated as follows: %OD = (Sample–PBS)/(AAPH–PBS) × 100.

### 2.7. NO Inflammatory and Anti-Inflammatory Activity in RAW 264.7 Cells

The ability of each honey sample to induce NO formation, a proinflammatory effect in RAW 264.7 macrophages (no LPS/IFN-*γ* added), was determined, and then, in a second experiment, the NO scavenging activity, an anti-inflammatory effect, was determined in LPS/IFN-*γ*-stimulated RAW 264.7 macrophages as described by Malan et al. [31].

For both experiments, a 70 *μ*L aliquot of RAW 264.7 cells was plated at a cell density of 1.5 × 10^6^ cells/mL (final concentration 1 × 10^6^ cells/mL). To determine the ability of the honey samples to induce NO formation, 10 *μ*L of the 10% (*v*/*v*) honey, pH controls, or digests (final concentration 1%) was added to the wells containing the plated RAW 264.7 macrophages (no LPS/IFN-*γ* added). To determine the NO scavenging activity, the RAW 264.7 cells were stimulated with 10 *μ*L of 1 *μ*g/mL *E. coli* LPS (final concentration 0.1 *μ*g/mL) and 10 *μ*L of 250 U/mL IFN-*γ* (final concentration 25 U/mL), and then 10 *μ*L of the honey, pH controls, or digests was added, yielding a final volume of 100 *μ*L.

For both experiments, exposure was for 24 h at 37°C and 5% CO_2_, and then 50 *μ*L of the supernatant was used to determine NO levels with 50 *μ*L of the Griess reagent (1% (w/v) sulphanilamide and 0.1% N-(1-naphthyl)-ethylenediamine dihydrochloride (NED) (w/v) in 2.5% phosphoric acid), and all data was reported as *μ*M nitrite equivalents (NE).

The viability of the attached RAW 264.7 macrophages was determined with the (3-[4,5-dimethylthiazol-2-yl]-2,5 diphenyl tetrazolium bromide) (MTT) assay. To the remaining media, 5 *μ*L of 1 mg/mL MTT solution was added and incubated for 3 h at 37°C and 5% CO_2._ The formazan formed was solubilised with 25% DMSO in ethanol, and the absorbance was measured at 570 nm and reported as % cell viability.

### 2.8. Nitric Oxide Scavenging Activity

To determine if the observed effect in the RAW 264.7 cell line was due to direct NO scavenging, the NO scavenging assay was undertaken. In this assay, the ability of the undigested (UD), GD, and GDD to scavenge sodium nitroprusside- (SNP-) generated NO was determined. To an 80 *μ*L volume of 5 mM sodium nitroprusside (SNP) in 0.1 M PBS that generates 22.91 *μ*M NO, 20 *μ*L of a 10% (*v*/*v*) honey solution was added. The levels of NO were measured after 1 h, by adding 100 *μ*L Griess reagent as prepared above, and after 10 min, the absorbance was read at 550 nm and reported as *μ*M NE.

### 2.9. Data Management and Statistical Analysis

All experiments were done at least three times in triplicate yielding 9 data points and presented as mean ± SEM. Statistical analysis was first done by determining the normality of data using the D'Agostino Pearson test and confirmed using the Shapiro-Wilk test. Depending on the normality of the data, significance differences were determined using one-way ANOVA followed by the Tukey post hoc test for data with a normal data set or the Kruskal-Wallis ANOVA followed by Dunn's post hoc test for data with a skewed data set. Significant differences were determined at *p* < 0.05.

## 3. Results

Honey characteristics were determined according to Serem and Bester [23] and Magoshi [32]. For the MAN (UMF15+) used in this study, the pH, Fru : Glu ratio, protein and proline content, H_2_O_2_ levels, and colour were 3.92, 0.80, 27.79 mg/100 g, 75.69 mg/100 g, 0.97 *μ*mol/mL, and 7.27 (AU). For the 6 FB honeys, the average pH, Fru : Glu ratio, protein and proline content, H_2_O_2_ levels, and colour were 4.28 ± 0.03, 0.75 ± 0.02, 35.37 ± 3.35 mg/100 g, 62.41 ± 2.38 mg/100 g, 1.08 ± 0.06 *μ*mol/mL, and 3.47 ± 0.05 (AU). All parameters were similar except that the MAN (UMF15+) was darker indicating a higher polyphenol content, confirmed in Table 1.

The TPC range of the FB honeys was 12.67 ± 2.07–74.49 ± 4.19 mg GAE/100 g (Table 1) with MAN (UMF15+) having a significantly higher TPC of 119.42 ± 13.94 mg GAE/100 g than all Fynbos honeys with significant differences ranging from *p* < 0.05 to *p* < 0.00001. This trend was the same for GD, and following GDD, the TPC of FB1 and FB6 was similar to MAN (UMF15+). The TPC of UD compared with GD was unchanged for all honeys, but compared with GDD, it was reduced for FB2 (*p* < 0.01) and FB5 (*p* < 0.05). Differences between GD and GDD were significant for MAN (UMF 15+), FB1, and FB2 (*p* < 0.01). For each honey comparing undigested samples to samples adjusted to pH 2 and pH 7, no significant changes were observed.

Antioxidant activity was measured with the TEAC and ORAC assays. For the TEAC assay, the range of the FB honeys was 5.46 ± 0.98–19.35 ± 1.23 *μ*mol TE/g (Table 1) with MAN (UMF15+) having 22.09 ± 1.29 *μ*mol TE/g. Comparing undigested Fynbos honeys with undigested MAN (UMF15+) honey, the antioxidant activity of FB2 (*p* < 0.05), FB3 (*p* < 0.01), FB4 (*p* < 0.0001) and FB5 (*p* < 0.001) was significantly less. Following GD, the antioxidant activity of all FB honeys was similar to MAN (UMF15+) except for FB4 (*p* < 0.0001) which was considerably less. Following GDD, FB2 (*p* < 0.01), FB3 (*p* < 0.05), and FB4 (*p* < 0.0001) were significantly lower than MAN (UMF15+), while the activity of FB1, FB5, and FB6 was similar. For UD vs. GD, antioxidant activity was unchanged for all honeys and was also unchanged for all honey for UD vs. GDD, except for FB2 where the antioxidant activity was decreased (*p* < 0.01). For GD vs. GDD, activity was significantly reduced for FB2 (*p* < 0.01). Changes in pH had no effect on the antioxidant activity. For the ORAC assay, the range for the FB honeys was 20.74 ± 2.78–63.66 ± 8.22 *μ*mol TE/g (Table 1), with MAN (UMF51+) having 48.41 ± 14.11 *μ*mol TE/g. No significant differences were found between MAN (UMF15+) and any of the FB honeys, for UD, GD, and GDD, and in addition, digestion did not alter the antioxidant measured with the ORAC assay.

For FB honey, the correlation between the phase of digestion for each assay and between assays was strong, and for TPC with UD vs. GD, UD vs. GDD, and GD vs. GDD, the correlation was 0.867, 0.958, and 0.918, respectively. The TEAC assay for UD vs. GD, UD vs. GDD, and GD vs. GDD was 0.990, 0.878 and 0.913, respectively. Lastly, for the ORAC assay, UD vs. GD, UD vs. GDD, and GD vs. GDD were 0.837, 0.920 and 0.905, respectively. The average %BA of the FB honey was 87.1 ± 27.0%, 78.0 ± 13.4%, and 100.7 ± 28.4% for the TPC, TEAC, and ORAC assays, respectively.

**Figure 1:**
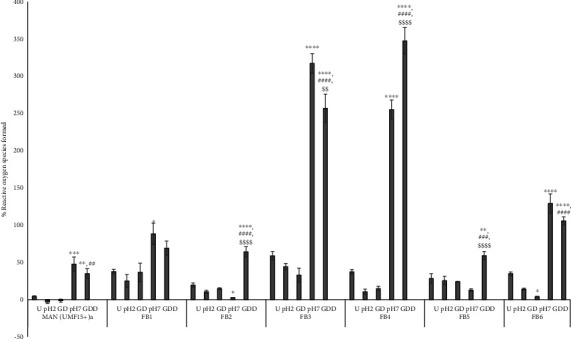
The cellular effects related to reactive oxygen species modulation of MAN (UMF15+) and FB1-FB6 honeys. Statistical analysis: ∗U vs. pH 2, GD, pH 7, and GDD, ^#^GD vs. GDD and ^$^pH 7 vs. GDD. Statistical relevance: symbols, one <0.05, two <0.01, three <0.001, and four <0.0001.

The effect of honey on ROS formation in a cellular model was determined in the Caco-2 cell line representative of the gastrointestinal tract. All UD honey samples caused a significant reduction in AAPH-mediated ROS formation from 100% to 4.28 ± 0.78% for MAN (UMF 15+) with a range of 19.50 ± 2.76–58.83 ± 5.70% for FB honey (Figure 1). Except for FB2, the ROS scavenging activity was significantly less than MAN (UMF15+) with *p* values ranging from *p* < 0.05 to *p* < 0.0001. For GD, differences between MAN (UMF15+) compared with the FB honeys were only significant for FB1 (*p* < 0.05). Transition from a low pH to a neutral pH environment associated with GDD resulted in a significant loss of this ROS scavenging effect. Compared with the control, although some ROS scavenging was still observed for MAN (UMF 15+), for FB1, FB2, FB5, and FB6, the ROS formed was similar to AAPH (*p* > 0.05), and for FB3 and FB4, a strong prooxidant effect (higher than AAPH) was observed, *p* < 0.0001. For the GDD phase of digestion, the correlation for the assays, TPC vs. TEAC, was 0.978; TPC vs. ORAC was 0.821; TPC vs. CAA was -0.666; TEAC vs. ORAC was 0.780; TEAC vs. CAA was -0.644; and ORAC vs. CAA was -0.840.

The pro- and anti-inflammatory effects related to NO induction or scavenging, respectively, were then determined. The ability of 1% honey, pH controls, and digests to induce NO was determined in RAW 264.7 cells following 24 h exposure. The RAW 264.7 murine macrophages alone, no LPS/IFN-*γ* added, did not produce NO, and the samples did not contain significant levels of nitrite measured with the Griess assay (Table 2 (a)). For undigested, MAN (UMF15+) and FB honey NO levels were similar and low, but following gastric and gastroduodenal digestion, they increased. These increases were significant for gastroduodenal digested MAN (UMF15+) (*p* < 0.05) and FB1 following gastric (*p* < 0.01) and gastroduodenal (*p* < 0.05) digestion. The greatest induction of NO levels was for the FB1 and FB3 after gastric and gastroduodenal digestion. In these experiments, the levels of NO-induced are similar to the 14.34 *μ*M, NO induced by 10 *μ*L of 1 *μ*g/mL *E. coli* LPS and 10 *μ*L of 250 U/mL IFN-*γ*. Compared to this control (14.34 *μ*M), MAN (UMF15+), FB5, FB6, and the undigested FB1, FB2, and FB3 have no proinflammatory effects. The GD and GDD samples of FB1, FB2, FB3, and FB4 have strong proinflammatory properties related to the induction of NO in RAW 264.7 murine macrophages.

Honey has well-described anti-inflammatory activity, and these effects were further evaluated in the RAW 264.7 murine macrophage/LPS/IFN–*γ* model. Undigested, MAN (UMF15+) had the best anti-inflammatory activity, with the Fynbos honeys having similar activity. Solutions of 1% undigested samples of MAN (UMF15+) (*p* < 0.01) and the gastric and gastroduodenal digests of FB5 (*p* < 0.001, *p* < 0.01) and FB6 (*p* < 0.01, *p* < 0.05) effectively scavenged NO (Table 2 (b)). Compared with undigested honey, NO scavenging was significant for MAN (UMF15+) after gastroduodenal digestion (*p* < 0.05) and FB5 after gastric digestion (*p* < 0.05). These results confirm the anti-inflammatory activity of undigested MAN (UMF15+) and identify FB5 and FB6 as honeys with strong anti-inflammatory activity postdigestion. The pH 2 and pH 7 samples had similar activity to the GD and GDD samples, respectively. In both the proinflammatory and anti-inflammatory experiments, the 1% samples were not cytotoxic as determined with the MTT assay (data not shown).

Anti-inflammatory activity related to a reduction in NO levels in the RAW 264.7 murine macrophage/LPS/IFN–*γ* model can be the result of direct NO scavenging or iNOS inhibition. The ability of the samples to reduce NO formed from SNP as a measure of direct NO scavenging was then determined (Table 2 (c)). Undigested MAN (UMF15+) (*p* < 0.0001), FB1 (*p* < 0.01), FB2 (*p* < 0.05), and FB6 (*p* < 0.05); the gastric digests of MAN (UMF15+) (*p* < 0.0001), FB1 (*p* < 0.01), and FB6 (*p* < 0.01); and all the gastroduodenal digests effectively scavenged NO (*p* < 0.05–*p* < 0.001). The best NO scavenging activity was seen in the undigested MAN (UMF15+) and FB1 samples. Following GD, all honeys had similar scavenging NO activity that was increased following GDD. This confirms that the anti-inflammatory effects of MAN (UMF 15+) are due to NO scavenging but also identifies FB1, FB2, and FB6 as honeys with strong direct NO scavenging activity.

## 4. Discussion

In this study, the effects of GIT digestion on the antioxidant and inflammatory properties of Fynbos honey were determined and compared with manuka honey. All parameters were similar except that MAN(UMF15+) was darker, and a higher polyphenol content was confirmed. The TPC of MAN (UMF15+) was 119.42 ± 13.94 mg GAE/100 g, within the range of 63.85 mg GAE/100 g, 103.99 mg GAE/100 g, 217 mg GAE/100 g, and 203 mg GAE/100 g reported for Comvita®, New Zealand [33], manuka MGO 250 and 400 [34], and Pure Gold Active 18+ manuka honey [35]. For all FB honeys, the TPC of each honey was significantly less than that of MAN (UMF15+).

In the present study, gastric and duodenal digestion of 0.5 h and 1 h, respectively, significantly reduced the TPC of MAN (UMF15+) to a %BA of 68.8%. O'Sullivan et al. [7] reported no significant change in the TPC of MAN (UMF 5+), following a simulated 1 h gastric and 2 h duodenal digestion. In contrast, Cianciosi et al. [36] reported that after gastric and subsequent duodenal digestion of 2 h each, the TPC of MAN (UMF15+) was reduced from 1.270 to 0.203 mg GAE/g (%BA of 15.98%). Differences in the reported %BA between studies can be attributed to the digestion times. In humans, Koziolek et al. [37] evaluated the transient times under fasted-state conditions. For the stomach, this was 7–202 min (median = 30 min), and for the small intestine, it was 67–533 min (median = 247 min); therefore, digestion times used in the present and other *in vitro* studies are within range and still physiologically relevant.

For Fynbos honey subjected to simulated gastrointestinal digestion for the TPC, the %BA was 87.1 ± 27.0%, and differences compared with MAN (UMF15+) were significant for FB2, FB3, FB4, and FB5. In contrast, the reported TPC of Irish, Tesco, and Lidl honey was unchanged after gastrointestinal digestion [7]. However, Seraglio et al. [2] reported that for three honeydew blends, the TPC as %BA was reduced to 74.18%, 65.74%, and 41.06% for the Urupema, Urubici, and Lages blends. Besides digestion times, factors further contributing to variability are the source, processing, polyphenol composition, and honey type.

To understand the complex antioxidant profile of food and derived products, several techniques are often used [38], and for antioxidant activity, these include the single electron transfer assays such as the TEAC, 2,2-diphenyl-1-picrylhydrazyl (DPPH), and ferric reducing antioxidant power (FRAP) assays. In addition, the inclusion of the hydrogen atom transfer-based, ORAC assay in the assessment of antioxidant activity is imperative when considering the physiological relevance of findings.

Anand et al. [39] reported TEAC values of 30.72 ± 0.27 and 21.28 ± 0.14 *μ*mol TE/g and ORAC values of 24.82 ± 0.5 and 12.40 ± 0.3 *μ*mol TE/g for MAN (UMF22+) and super manuka honey, respectively. In the present study for MAN (UMF15+), the antioxidant activity was similar and was 22.09 ± 1.29 *μ*mol TE/g. In contrast, the ORAC value of 48.41 ± 14.11 *μ*mol TE/g was higher. The antioxidant activity of FB1 and FB6 was similar to MAN (UMF15+) but was reduced for FB2, FB3, FB4, and FB5 when measured with the TEAC assay, while with the ORAC assay, the antioxidant activity of all FB honey was similar to MAN (UMF15+).

With digestion, the %BA and TEAC assays for MAN (UMF15+) were reduced to 77.9%. Other studies have also reported a reduction in the antioxidant activity of manuka honey following digestion [7, 36]. The antioxidant activity of MAN (UMF5+) was significantly reduced when measured with the FRAP and DPPH assays [7]. Likewise, %BA for MAN (UMF15+), evaluated with the FRAP, TEAC, and DPPH assays, was reduced to 45.74, 45.52, and 13.66%, respectively [36]. With the hydrogen atom transfer-based ORAC assay, the %BA for MAN (UMF15+) was increased to 121.20% indicating that with digestion, inhibitors of peroxyl radicals are released such as matrix-bound polyphenols or antioxidant peptides [40].

The antioxidant activity of the FB honeys measured with the TEAC and ORAC assays following digestion resulted in a %BA of 78.2% and 100.7%, respectively. In the study by O'Sullivan et al. [7], the antioxidant activity, FRAP assay, was reduced only for Tesco honey, while with the DPPH assay, the % radical scavenging was reduced for all honeys. *In vitro*, gastrointestinal digestion reduced the %BA of an organic Necta® Floral, phloem honey measured with the TEAC assay to 20.17% [8]. The %BA for the Urupema, Urubici, and Lages honeydew blends was 74.92%, 90.16%, and 92.16%, respectively, for the FRAP assay and 100.96%, 88.97%, and 78.6%, respectively, for the DPPH assay [2]. The measured effects of digestion on the antioxidant activity of honey depend on the assay and type, where honeydew blends are less affected by digestion. For FB honey and digests, a strong correlation was found between the phases of digestion and between the TPC, TEAC, and ORAC assays.

The degree of polyphenol stability in the different phases of digestion is pH dependent. In general, polyphenols are stable at low pH associated with gastric digestion while some polyphenols are unstable at neutral or alkaline pH, associated with duodenal digestion. Under these conditions, susceptible polyphenols undergo degradation or transformation, forming molecules with altered chemical and biological properties [2].

For MAN (UMF15+), following digestion, Cianciosi et al. [36] reported that the %BA of the phenolic acids was 100%, and the flavonoids were 0.86% resulting in a %BA of 39.8% for MAN (UMF15+). Likewise, Mannina et al. [6] reported that following the digestion of MAN (UMF25+), the levels of the phenolic acids that were specific markers for manuka honey were unchanged.

In the present study, pH alone had little effect on the TPC, and antioxidant activity measured with TEAC and ORAC assays. Only for pH 2 vs. GD for FB1 was there a significant increase in TPC, and for pH 7 vs. GDD for FB2, a significant decrease in TPC was found that did not translate into a significantly altered antioxidant activity. Like the study of Seraglio et al. [2], in the present study, the Folin-Ciocalteu reducing capacity or TPC assay was the most sensitive in measuring changes in antioxidant properties.

In contrast to an unaltered or reduced antioxidant activity, for *Mimosa scabrella* Bentham honeydew, the concentration of individual polyphenols determined with LC-ESI-MS/MS was increased [2]. Following duodenal digestion, the sum of the individual phenolic compounds was increased from 575.74, 704.47, and 1850.34 to 696.85, 51.63, and 2713.78 *μ*g/100 g for Urupema, Urubici, and Lages honey blends, respectively. The differences found between measured antioxidant activity, compared with LC-ESI-MS/MS analysis, were proposed to be due to the complexity of honey, where in addition to polyphenols, other compounds, such as vitamins and peptides, also contribute to the antioxidant properties of honey. pH effects and proteolysis can reduce the antioxidant activity of the antioxidant vitamins and peptides, respectively. For example, the antioxidant vitamin, vitamin C, is pH sensitive, and Costa et al. [8] reported that for Necta® Floral, phloem honey, the %BA for vitamin C was reduced to 46.96% following gastroduodenal digestion.

To further validate these results, the ability of the honey samples to protect Caco-2 cells from AAPH-generated peroxyl radical damage was determined. This cellular model provides a more physiologically relevant assessment of these properties [41]. The DCFH-DA assay represents the ORAC assay in a cellular environment, where AAPH is also used to generate peroxyl radicals. Undigested and gastric digested, MAN (UMF15+) and FB honeys effectively protected Caco-2 cells against oxidative damage. Following gastric digestion, the scavenging activity of FB6 was significantly increased. This confirms the ability of honey to protect the gastric mucosa against ROS-induced ulceration [16, 17].

In contrast with duodenal digestion, a loss of peroxyl scavenging activity was observed, especially for FB3 and FB4, where ROS levels were increased. The latter is attributed to the pH-dependent autoxidation of flavonoids and is reported to play a role in the anticancer effects observed following honey digestion. In the Caco-2, colonic cancer cell line, a significant loss in cell viability was observed at 2.5 to 7.5 mg/mL undigested and 1 to 3 mg/mL for digested manuka honey, indicating that with digestion cytotoxicity is increased [7]. Cianciosi et al. [36] compared the effect of undigested with digested MAN (UMF15+) on the viability of HCT-116, colon cancer cells. The IC_50_ for the undigested and digested honey was 16.97 mg/mL (equivalent to 1.15% manuka honey, density 1.47 g/mL) [35] and 14.32 mg/mL (0.97% honey), respectively, after 72 h exposure that indicates an increase in cytotoxicity. Additional anticancer effects following digestion were the inhibition of colony formation and apoptosis, via a cell cycle block in the S and SubG1 phases for the undigested and digested MAN (UMF 15+) honey. In a further study, Cianciosi et al. [36] with HCT-116 spheroids reported that manuka honey (UMF15+), both digested and undigested, induced apoptosis and intracellular ROS accumulation, decreased expression of the ATP-binding cassette (ABC) transporter, and downregulation of the Wnt/*β*-catenin pathway.

Besides these studies, further anticancer properties of manuka honey have been extensively reviewed by El Senduny et al. [42]. Manuka honey has identified anticancer activity in murine melanoma (B16.F1), colorectal carcinoma (CT26), breast (MCF-7 and MDA-MB-231), lung (A549), and colon (HCT-116 and LoVo) cancer cell lines [42]. The molecular mechanisms of several types of honey evaluated in various cell lines, species, and models were reviewed by Talebi et al. [43]. Clearly, manuka honey has anticancer effects [44]; likewise, other honey types, such as Fynbos honey, potentially also have beneficial anticancer properties.

In neutral or alkaline solutions as well as cell culture media, susceptible flavonoids undergo autoxidation, a pH-dependent process that occurs rapidly at alkaline pH, and likewise in the intestines, pH would also contribute to autoxidation [45]. Polyphenol autoxidation in cell culture media, pH 7.4, results in H_2_O_2_ formation [46]. Grzesik et al. [45] reported that in PBS, pH 7.4, 127.2 ± 4.7 *μ*M, 116.7 ± 3.3 *μ*M, and 110.2 ± 2.0 *μ*M H_2_O_2_ formed for 1 mM propyl gallate, epigallocatechin gallate (EGCG), and quercetin, respectively. In DMEM/F12, H_2_O_2_ levels were lower with the formation of 95.2 ± 1.9 *μ*M, 90.2 ± 2.6 *μ*M, and 76.4 ± 5.9 *μ*M H_2_O_2_ for the same concentration of propyl gallate, EGCG and quercetin, respectively. The presence of amino acids, vitamins, and other molecules in cell culture media with antioxidant activity accounts for the lower H_2_O_2_ levels for DMEM/F12 compared with PBS. The kinetics of H_2_O_2_ formation was linear, 0–60 min reaching a plateau after 120 min. Concentrations of 80 *μ*M propyl gallate, 100 *μ*M EGCG, and 40 *μ*M quercetin reduced the viability of DU-145 cells after 24 h exposure [45]. Formation of ROS from H_2_O_2_ via the Fenton reaction requires transition metals that catalyze the conversion of H_2_O_2_ to ROS that accumulates in cell culture causing cell death. Addition of iron chelators to the cell culture media reduced the effects of H_2_O_2_ [45]. Transition metals found in honey that can act as catalysts of the Fenton reaction include Al, Co, Cu, Fe, Mn, Ni, and Pb [2]. The %BA of minerals, including several transition metals, following gastroduodenal digestion for different types of Polish, Brazilian blossom, and Brazilian and Portuguese blossom honeys was 73-100%, 93-220%, 74-107%, and 4-25%, respectively [2]. This indicates that, with digestion, these metals can catalyse the Fenton reaction with subsequent ROS formation.

Manuka honey contains MG, and although cytotoxic [42], findings indicate that following digestion, MG levels are reduced due to GOx binding [47] or binding to the digestive enzymes [26]. In addition, different types of honey also contain variable H_2_O_2_ levels formed via glucose oxidase (GOx) mediated conversion of glucose to gluconolactone with the reduction of molecular oxygen to H_2_O_2_. Levels of H_2_O_2_ are increased following the dilution of honey due to the activation of the GOx system [47]. In an acidic environment, in the presence of transition metals [2, 48] increased ROS formation occurs and subsequently, an associated loss of cellular antioxidant activity. However, in this acidic environment, polyphenols are stable and can effectively scavenge H_2_O_2_. Dźugan et al. [49] found that Polish buckwheat honeys, with a H_2_O_2_ content of 50 ± 2 to 1100 ± 100 *μ*M, had significant levels of antioxidant activity (DPPH and FRAP assays) with p-hydroxybenzoic acid, p-coumaric acid, and kaempferol being the most abundant. These researchers also concluded that the antibacterial activity of these honeys was due to the presence of these phenolic acids rather than the high H_2_O_2_ content. Likewise, the anticancer effect of honey is not limited only to H_2_O_2_ formation; in addition, specific polyphenols contribute significantly to this effect. Cianciosi et al. [36] showed that in manuka honey, the phenolic acid composition was unchanged, but the flavonoid levels were reduced; generally, the assumption is made that with autoxidation, antioxidant activity is lost. However, studies on the autoxidation of EGCG have led to the identification of theasinensin A, theasinensin D, and oolongtheanin digallate with an EGCG equivalent antioxidant capacity (EEAC) of 1.59, 1.92, and 1.54, respectively [50]. It was concluded that the autooxidation products of polyphenols may not necessarily be inactive, following H_2_O_2_ formation, and have antioxidant activity better than that of the parent compound. Consequently, the H_2_O_2_ levels generated are dependent on honey type, polyphenol composition, including structure and concentration, transition metals, and the activity of the formed autoxidation products. For FB honey, pH 7 was associated with an increase in ROS formation for FB1, FB3, FB4, and FB6 but not FB2 and FB5, indicating that pH effects are complex and are a combination of H_2_O_2_ formation, activity of pH-stable phenolic acids, and the activity of the autoxidation products.

In the GIT, endogenous NO (eNOS) released from the gastric mucosa, vascular endothelium, and sensory neurons, together with the prostaglandins, maintain the integrity and microcirculation of the gastric mucosa. In addition, NO can increase the resistance of the GIT mucosa to injury, and the development of NO-releasing nonsteroidal anti-inflammatory drugs (NSAIDs) reduces the risk for NSAID-induced ulcer development [19]. A proinflammatory response is an important component of cancer immunology and involves macrophage stimulation and release of mediators with tumoricidal activity. In established cancers, high NO promotes processes that activate immunity and improve the efficacy of chemotherapy [51]. However, the role of NO in cancer development is more complex, where high NO influx leads to DNA damage, p53 activation, and nitrosactive stress which may initially promote carcinogenesis. For example, *Helicobacter pylori* infections increase the expression of iNOS and, together with oxygen radicals, form the highly reactive peroxynitrite radical, that is cytotoxic and induces inflammation, associated with an increased risk for cancer [20]. Therefore, the role of honey in this process following digestion is an important consideration.

MAN(UMF15+) honey did not induce NO formation, but with digestion, NO levels were increased, indicating that with digestion, MAN (UMF15+) in the duodenum is proinflammatory. A similar effect was observed for only FB1, where with digestion, NO levels were increased. Tonks et al. [52] reported that although 1% solutions of manuka, pasture, and jelly bush honey had a LPS content of 0.056, 0.340, and 0.690 ng/mL, increased levels of TNF-*α*, IL-1*β,* and IL-6 induced in MM6 and human monocytes were unrelated to LPS levels. Subsequently, Tonks et al. [53] identified that increased cytokine production in human monocytes or MM6 cells was due to type II arabinogalactan proteins. Raynaud et al. [12] also confirmed that LPS alone, at a concentration of 94.2 ± 3.1 ng/g in thyme honey, did not significantly contribute to AP-1 and NF-𝛋B activation and cytokine production in RAW 264.7 macrophage cells exposed to 1.28%-2,56% (*v*/*v*) honey. Other immune modulatory proteins identified in honey that increased TNF-*α* in monocytes, macrophages, and keratinocytes were the peptides, apalbumin-1 (Apa1) and -2, major royal jelly, and apisimin [54]. Partial tryptic digestion of Apa1 resulted in fragments, and especially the N-terminal fragment increased proinflammatory TNF-*α* levels in murine macrophages when compared with the other fragments, Apa1 and recombinant Apa1 [55].

TNF-*α*, IL-1*β*, and LPS are the main inducers of iNOS, that catalyzes the formation of NO, a critical inflammatory response of the innate immunity [56]. M1 macrophages induced by Th1 cytokines including INF-𝛾 and LPS lead to the production and the secretion of high levels of the proinflammatory cytokines, TNF-*α*, IL-1*α*, IL-1*β*, IL-6, IL-12, IL-23, and COX-2 [57]. These proinflammatory cytokines eradicate pathogens, and this process is mediated by the activation of the nicotinamide adenine dinucleotide phosphate (NADPH) oxidase system with subsequent ROS generation [57] and the formation of nitrosative species. In excess, these species can cause tissue damage, and subsequently, scavenging of ROS and NO limits the formation of nitrosative species, ensures rapid tissue recovery, and prevents the development of associated pathology.

MAN (UMF15+) effectively reduced NO levels in the LPS/IFN-*γ* RAW 264.7 macrophage model, but with gastric digestion, NO scavenging was reduced, and with duodenal digestion, NO was lost. In contrast for FB5 and FB6, with digestion, NO scavenging was increased, with FB6 having the greatest NO scavenging effect. This effect is either due to the direct NO scavenging or iNOS modulation. Direct NO scavenging was then determined with the SNP assay. Undigested and gastric digested MAN (UMF15+), FB1, and FB6 honey directly scavenged NO. Following gastroduodenal digestion, all honey digests directly scavenged NO, although this effect was not observed in the LPS/IFN-*γ* RAW 264.7 macrophage model.

A finding not previously reported is that honey can have a pro- or anti-inflammatory effect depending on the honey and the physiological environment. In the RAW 264.7 macrophages, in the absence of proinflammatory molecules, NO can be induced and depending on levels may have either a protective or inflammatory effect. In contrast in the RAW 264.7 macrophages, where NO is induced by LPS/IFN-*γ*, NO scavenging can occur. In the present study, findings were that MAN (UMF15+) is predominantly a honey with anti-inflammatory properties due to direct NO scavenging and supports the findings of previous studies [58]. However, with digestion, this effect is reduced, and a proinflammatory effect is observed, while in contrast, the anti-inflammatory activity of FB5 and FB6 increases.

Indomethacin is widely used to induce gastric ulcers in experimental animal models [59]. The proposed mechanism is the inhibition of cyclooxygenase-1 (COX-1), prostaglandin E2, (PEG2), bicarbonate, and mucus, thereby reducing the protective effects of these molecules in the GIT [59]. Many *in vivo* studies have identified the ability of honey to reduce indomethacin as well as ethanol and acidified ASA-induced gastric lesions due to the anti-inflammatory activity of honey and the associated reduction of NO, ROS, and associated nitrosative species of honey [60]. In this study, in the gastric phase of digestion, MAN (UMF15+) and the FB honeys have antioxidant and NO scavenging activity, indicating that MAN (UMF15+) has the potential to reduce the levels of nitrosative species. With gastroduodenal digestion, the antioxidant activity of MAN(UMF15+) is retained, although both ROS and NO scavenging are reduced, indicating that although reduced, some scavenging of nitrosative species can occur. In general, for FB honey, for undigested and gastric digested honey, antioxidant activity is retained, and for undigested honey, pro- and anti-inflammatory activity is absent while the cellular anti-inflammatory activity of FB5 and FB6, and the NO scavenging activity of FB1 and 6 is increased following gastric digestion. This indicates that FB honey has the potential to reduce nitrosative levels but not as effectively as MAN (UMF15+).

In the duodenal phase of digestion, the activity of MAN (UMF15+) is retained, although the cellular anti-inflammatory activity is lost, and some NO scavenging activity is retained. In contrast for FB honey, in the duodenal phase of digestion, the antioxidant effect is reduced, although not associated with significant changes in antioxidant activity when evaluated with noncellular assays. Increased ROS formation has been identified as one of the anticancer mechanisms of several types of honey [7, 8] and was observed especially for FB3 and FB4. With gastroduodenal digestion, FB1 had a strong proinflammatory while FB5 and FB6 had strong anti-inflammatory effects. The molecules and mechanisms associated with these differences are the focus of future studies.

## 5. Conclusion

Fynbos honey has antioxidant activity that is unaffected with gastric digestion, but with gastroduodenal digestion, there is a variable loss of polyphenol content and antioxidant activity, TEAC assay, although activity measured with the ORAC assay is unaltered. Evaluation of CAA in the Caco-2 cell line revealed a strong prooxidant effect after gastroduodenal digestion, which was pH dependent for FB1, FB3, FB4, and FB6 but not FB2 and FB5. This identifies FB honey, especially FB3 and FB4, as honeys with potential anticancer properties, related to ROS production, better than MAN (UMF 15+) honey.

In murine macrophage (RAW 264.7) cells, all honeys induced different levels of NO which was significantly increased with digestion for MAN (UMF15+) and FB1. In LPS/IFN-*γ*-stimulated RAW 264.7 macrophages, only undigested MAN (UMF15+) effectively reduced NO levels, and with digestion, NO scavenging activity of MAN (UMF15+) was reduced but increased for FB5 and FB6. In a noncellular environment, MAN (UMF15+), FB1, FB2, and FB6 effectively scavenged NO to a variable degree following gastric digestion but strongly for all honeys after gastroduodenal digestion. This study has identified that undigested and gastric-digested FB honey has antioxidant properties with potential anticancer effects following gastroduodenal digestion, related to ROS formation. MAN (UMF15+) had anti-inflammatory effects which were lost postdigestion (although direct NO scavenging activity is present), and in contrast, FB5 and FB6 gained anti-inflammatory effects postdigestion. Although further elucidation of the mechanisms involved is required, this study shows that FB honey has potential health benefits when digested.

## Figures and Tables

**Table 1:** Polyphenolic composition and the antioxidant activity of MAN UMF15+ and FB1-FB6 honeys.

Sample	UD	pH 2	GD	pH 7	GDD	BA (%)
TPC (mg GAE/100 g)						
MAN (UMF15+)	119.42 ± 13.94	150.56 ± 9.88	161.65 ± 9.56	106.89 ± 7.38	81.94 ± 7.75^##^	68.6%
FB1	74.49 ± 4.19^∗^	60.85 ± 2.89	100.67 ± 5.24^∗∗^^, ††^	74.60 ± 3.68	58.26 ± 11.61^##^	78.2%
FB2	53.98 ± 1.70^∗∗∗^	43.80 ± 2.22	54.88 ± 1.35^∗∗∗∗^	49.19 ± 2.52	36.08 ± 3.85^∗∗^^, $$, ##, †^	66.8%
FB3	49.59 ± 4.98^∗∗∗^	43.09 ± 3.27	56.24 ± 1.64^∗∗∗∗^	45.45 ± 1.26	44.01 ± 3.45^∗^	88.6%
FB4	12.67 ± 2.07^∗∗∗∗^	14.43 ± 4.55	23.53 ± 10.61^∗∗∗∗^	17.98 ± 5.54	17.81 ± 4.89^∗∗∗^	140.6%
FB5	59.16 ± 5.48^∗∗^	46.73 ± 2.23	45.40 ± 1.05^∗∗∗∗^	45.11 ± 2.65	41.95 ± 4.49^∗^^$^	70.9%
FB6	71.90 ± 11.80^∗∗^	58.80 ± 11.10	74.83 ± 14.87^∗∗∗∗^	60.00 ± 8.22	56.02 ± 8.41	77.9%
MEAN ± SEM^&^	53.62 ± 12.91	44.17 ± 16.63	59.26 ± 15.19	57.03 ± 27.88	42.36 ± 8.51	87.1 ± 27.0%

TEAC assay (*μ*mol TE/g)						
MAN (UMF15+)	22.09 ± 1.29	21.64 ± 1.06	21.54 ± 0.97	17.69 ± 1.78	17.21 ± 0.52	77.9%
FB1	19.35 ± 1.23	18.65 ± 1.47	21.28 ± 1.01	16.79 ± 1.59	15.98 ± 1.57	82.6%
FB2	16.90 ± 1.03^∗^	15.88 ± 1.60	17.10 ± 1.31	12.00 ± 1.21	8.94 ± 0.87^∗∗^^, $$, ##^	52.9%
FB3	14.27 ± 0.26^∗∗^	15.51 ± 1.32	15.47 ± 2.02	12.70 ± 1.15	10.69 ± 0.81^∗^	74.9%
FB4	5.46 ± 0.98^∗∗∗∗^	5.65 ± 0.51	5.32 ± 1.34^∗∗∗∗^	3.82 ± 0.70	4.87 ± 1.44^∗∗∗∗^	89.2%
FB5	13.91 ± 0.73^∗∗∗^	16.37 ± 1.17	16.29 ± 1.61	12.94 ± 1.19	11.33 ± 1.08	81.5%
FB6	19.24 ± 1.10	20.36 ± 1.14	20.88 ± 1.07	17.45 ± 1.50	16.89 ± 1.14	87.8%
MEAN ± SEM^&^	14.85 ± 2.98	16.29 ± 4.85	16.06 ± 3.34	13.34 ± 4.48	11.45 ± 4.48	78.2 ± 13.4%

ORAC assay (*μ*mol TE/g)						
MAN (UMF15+)	48.41 ± 14.11	58.41 ± 8.64	53.88 ± 10.81	42.34 ± 4.19	58.67 ± 8.27	121.2%
FB1	59.09 ± 9.20	59.34 ± 16.34	59.43 ± 18.54	63.45 ± 17.42	54.47 ± 12.42	92.2%
FB2	43.07 ± 11.08	52.13 ± 12.64	58.08 ± 11.63	46.67 ± 16.58	52.45 ± 10.61	121.9%
FB3	39.21 ± 0.66	40.17 ± 8.20	45.79 ± 3.61	45.46 ± 5.78	50.37 ± 10.67	128.5%
FB4	20.74 ± 2.78	19.80 ± 6.94	20.16 ± 5.86	15.96 ± 4.30	10.17 ± 1.65	49.03%
FB5	63.66 ± 8.22	55.45 ± 3.31	50.80 ± 6.17	52.92 ± 6.80	65.89 ± 10.93	103.5%
FB6	60.08 ± 5.90	47.32 ± 9.70	66.59 ± 4.87	69.17 ± 5.09	65.59 ± 8.41	109.2%
Mean ± SEM^&^	47.64 ± 16.48	45.70 ± 14.32	50.14 ± 16.36	48.94 ± 18.65	49.82 ± 20.53	100.7 ± 28.4%

Data is an average of at least 3 experiments and represented as mean ± SEM. ^∗^ Significant differences of FB vs. MAN (UMF15+) for UD, GD, and GDD. ^$^ Significant differences of UD vs. GD and GDD. ^#^ Significant differences of GD vs. GDD. † Significant differences of pH 2 vs. GD and pH 7 vs. GDD. ‡ Significant differences of pH 2 vs. pH 7. Statistical analysis: symbols: one <0.05, two <0.01, three <0.001, and four <0.0001. ^&^ Calculated for FB only.

**Table 2:** The NO-mediated cellular pro- and anti-inflammatory effects of honey.

	UD	GD	GDD
(a) Cellular proinflammatory model: -LPS/IFN-*γ*			
MAN (UMF 15+)	0.21 ± 0.67	4.19 ± 0.62	7.93 ± 0.80^$^
FB1	2.48 ± 1.18	11.67 ± 0.50^$$^	10.73 ± 1.59^$^
FB2	2.46 ± 1.13	7.43 ± 0.56	7.79 ± 0.54
FB3	5.94 ± 2.41	14.92 ± 1.20	12.17 ± 1.76
FB4	7.60 ± 2.93	10.19 ± 1.64	8.28 ± 1.58
FB5	0.23 ± 0.57	−0.11 ± 0.60	0.40 ± 0.66
FB6	1.59 ± 0.87	2.26 ± 0.75	3.55 ± 0.72
Mean ± SD^&^	3.38 ± 2.80	7.73 ± 5.74	7.15 ± 4.43

(b) Cellular anti-inflammatory model: +LPS/IFN-*γ* (control: 14.34 *μ*M)			
MAN (UMF 15+)	8.62 ± 6.05^∗∗^	10.31 ± 1.60	15.95 ± 1.36^$^
FB1	11.83 ± 2.96	13.59 ± 1.49	15.82 ± 0.86
FB2	15.23 ± 3.81	13.87 ± 1.92	13.72 ± 3.01
FB3	15.58 ± 3.02	13.87 ± 1.92	13.72 ± 3.01
FB4	17.33 ± 3.38	9.25 ± 1.66	10.95 ± 3.49
FB5	15.41 ± 2.57	4.97 ± 1.00^∗∗∗^^, $^	6.44 ± 1.29^∗∗^
FB6	11.39 ± 2.87	3.10 ± 0.35^∗∗^	5.70 ± 1.03^∗^
Mean ± SD^&^ (% scavenging)	14.46 ± 2.34 (−0.84%)	9.78 ± 4.82 (31.83%)	11.06 ± 4.17 (22.89%)

(c) Direct NO scavenging: SNP assay: control (21.91 *μ*M)			
MAN (UMF 15+)	3.56 ± 0.70^∗∗∗∗^	2.75 ± 0.32^∗∗∗∗^	6.82 ± 1.08^∗∗∗^
FB1	9.87 ± 1.61^∗∗^	9.82 ± 1.35^∗∗^	6.49 ± 0.48^∗∗∗^
FB2	10.77 ± 2.07^∗^	12.84 ± 0.65	5.46 ± 0.88^∗∗∗^
FB3	13.81 ± 2.75	14.82 ± 1.17	9.72 ± 1.20^∗^
FB4	15.41 ± 2.76	16.68 ± 0.80	10.62 ± 1.13^∗^
FB5	15.57 ± 2.10	16.01 ± 1.14	9.47 ± 0.95^∗∗^
FB6	11.38 ± 2.23^∗^	7.67 ± 0.69^∗∗^	9.21 ± 0.78^∗∗^
Mean ± SD^&^ (% scavenging)	12.80 ± 2.46 (41.57%)	12.97 ± 3.59 (40.79%)	8.50 ± 2.04 (61.23%)

-LPS/IFN-*γ*: statistical analysis ^$^UD vs. GD or GDD. +LPS/IFN-*γ* (control: 14.34 *μ*M): statistical analysis ^∗^ vs. control; $UD vs. GD or GDD. SNP assay control: (21.91 *μ*M), statistical significance ^∗^ vs. control (21.91 *μ*M), $UD vs. GD or GDD. Symbols: one <0.05, two <0.01, three <0.001, and four <0.0001. ^&^ Calculated for FB only.

## Data Availability

Data is available on request from the corresponding author.
